# Functional characterization and mechanistic basis of antifungal and plant growth-promoting traits of *Streptomyces* sp. LZZY-S40

**DOI:** 10.3389/fmicb.2026.1800596

**Published:** 2026-04-15

**Authors:** Lan Ye, Pinjiao Jin, Zhenhua Liu, Dongmiao Qin, Haolin Wang, Liyang Chu

**Affiliations:** 1 School of Environmental and Food Engineering, Liuzhou Polytechnic University, Liuzhou, China; 2 Heilongjiang Academy of Black Soil Conservation and Utilization, Postdoctoral Station of Heilongjiang Academy of Agricultural Sciences, Harbin, China; 3 Yantai Key Laboratory of Characteristic Agricultural Bioresource Conservation and Germplasm Innovative Utilization, College of Life Sciences, Yantai University, Yantai, China

**Keywords:** antifungal activity, extracellular enzymes, genome analysis, metabolomics, plant growth promotion, streptomycetes

## Abstract

Biological control agents (BCAs) represent a sustainable and environmentally benign alternative to synthetic chemical pesticides in modern agriculture. Among these, *Streptomyces* species are widely recognized for their ability to suppress phytopathogens and promote plant growth through diverse functional traits. In this study, a *Streptomyces* strain designated LZZY-S40 (=CGMCC No.30320) was evaluated for its antifungal activity using dual-culture assays and for its plant growth-promoting (PGP) potential through seedling growth assays and functional trait analysis. Genome mining and LC–MS-based metabolomic profiling were further performed to investigate its biosynthetic potential and secondary metabolite composition. Strain LZZY-S40 exhibited broad-spectrum antifungal activity against nine plant-pathogenic fungi, with inhibition rates ranging from 55.8% to 92.8%, the strongest suppression was observed against *Exserohilum turcicum* (92.8%). Further analyses indicated that strain LZZY-S40 is capable of producing extracellular proteases, suggesting a potential contribution to its antifungal activity. Genome mining identified several secondary metabolite biosynthetic gene clusters (BGCs) associated with the production of antimicrobial compounds, including those responsible for the biosynthesis of undecylprodigiosin and manumycin A. These genomic predictions were corroborated by LC-MS analysis. In addition to its antifungal properties, strain LZZY-S40 significantly enhanced the growth of wheat seedlings. At a concentration of 10^5^ CFU·ml^−1^, it increased root length by 88.3%, shoot length by 37.9%, and fresh weight by 32.1%. Consistent with these phenotypic effects, the strain tested positive for multiple well-established PGP traits—including indole-3-acetic acid (IAA) production, siderophore secretion, 1-aminocyclopropane-1-carboxylate (ACC) deaminase activity, and nitrogenase-mediated nitrogen fixation—providing mechanistic support for its growth-enhancing capacity. Collectively, these findings demonstrate that strain LZZY-S40 exhibits robust antifungal activity against a broad spectrum of plant-pathogenic fungi and promotes plant growth significantly. Mechanistic investigations provided preliminary insights into the strain's antifungal and plant growth-promoting traits, contributing to a better understanding of its potential as a dual-action biocontrol agent.

## 1 Introduction

The growing challenges confronting modern agriculture, including the increasing prevalence of fungicide-resistant phytopathogens and the environmental impacts of chemical fungicides, have intensified the demand for sustainable disease control strategies (Zhuang et al., 2023; Luo et al., 2024; Aravindaram et al., 2025; Duraimurugan et al., 2025). Biological control agents (BCAs), especially microbial biocontrol agents, have gained prominence as viable substitutes for synthetic pesticides (Hu et al., 2026; Omokaro, 2026; Zhao et al., 2026; Zhou et al., 2026).

In this context, *Streptomyces* strains, soil-dwelling actinomycetes renowned for their diverse secondary metabolite production, have emerged as promising candidates for biocontrol (Chen et al., 2017; Li et al., 2026; Hu et al., 2026). These secondary metabolites include antimicrobial agents such as antibiotics and antifungal compounds, as well as extracellular enzymes that enhance their biocontrol efficacy (Newitt et al., 2019; Abdelaziz et al., 2026; Rai et al., 2026; Tran et al., 2026). In addition to their antimicrobial activity, *Streptomyces* strains also promote plant growth through multiple well-documented mechanisms. These include the biosynthesis of phytohormones—particularly indole-3-acetic acid (IAA) (Myo et al., 2019); production of 1-aminocyclopropane-1-carboxylate (ACC) deaminase, an enzyme that mitigates ethylene-mediated stress in plants (Ho et al., 2023; Suman et al., 2023; Chandwani et al., 2025); and facilitation of nitrogen fixation via symbiotic or associative interactions (Xia et al., 2017; AlMaroai, 2019). Several commercialized strains, including *S. lydicus* WYEC108, *S. violaceusniger* YED9, *S. griseoviridis* K61, and *S. saraceticus* K400, have already been developed as biofungicides due to in disease suppression and plant growth promotion (Sarwar et al., 2019; Wang et al., 2022; Chakraborty et al., 2023).

Despite their significant potential, the large-scale agricultural application of *Streptomyces* species remains constrained (Wang et al., 2021; Ferraiuolo et al., 2021; Nikky et al., 2021). Key limitations include the scarcity of diverse, commercially scalable strains and an incomplete understanding of the biological factors governing their biocontrol and plant growth-promoting (PGP) traits (Pawaskar and Kerkar, 2021; Abo-Elyousr et al., 2026; Erdinc and Cufaoglu, 2026; Fain et al., 2026). Although *Streptomyces* strains exhibiting antagonistic or plant growth–promoting properties have been reported, many studies remain largely descriptive and lack integrated physiological, genomic, and metabolic evidence to explain the biological basis of these functional traits. This limitation restricts a more comprehensive understanding of how these microorganisms exert their beneficial effects and hinders the rational identification and development of promising strains for agricultural applications (Hu et al., 2021; Alam et al., 2022).

In this study, *Streptomyces* sp. LZZY-S40 was investigated for its potential as a biological control agent. The strain demonstrated broad-spectrum antifungal activity against multiple phytopathogenic fungi and significantly enhanced the growth of wheat seedlings. To gain preliminary insights into the mechanisms underlying these functional traits, we combined phenotypic assays with enzymatic characterization, genome-based analysis, and metabolomic profiling to examine extracellular enzyme production, plant growth-related traits, secondary metabolite biosynthetic potential, and metabolite composition. This integrated analysis provides a more comprehensive characterization of the functional attributes of strain LZZY-S40 and contributes to a better understanding of the factors potentially supporting its antifungal and plant growth–promoting activities. These findings provide a foundation for evaluating the application potential of strain LZZY-S40 and may facilitate the identification of functionally similar strains for sustainable crop protection.

## 2 Material and method

### 2.1 Sample collection

Soil samples were collected from the rhizosphere of tea plants in Sanjiang County, Guangxi Zhuang Autonomous Region, China (25°51′11.51″ N, 109°30′59.55″ E). After collection, the samples were transported to the laboratory in sterile bags and subsequently air-dried at room temperature for 48 h.

### 2.2 Isolation and preservation of actinobacterial strain

Ten grams of dried soil was suspended in 90 ml of sterile water and shaken at 250 rpm for 30 min at 28 °C. The resulting suspension was serially diluted and spread onto Gause's synthetic agar No. 1 (containing 0.5% peptone, 0.3% beef extract, 0.5% NaCl, and 2% agar), supplemented with nystatin (50 mg/L) and nalidixic acid (20 mg/L). After incubation at 28 °C for 14 days, morphologically distinct colonies were selected, and purified by repeated streaking onto International *Streptomyces* Project (ISP) medium 3 (containing 0.4% yeast extract, 1% malt extract, 0.4% dextrose, and 2% agar). Purified isolates were preserved as glycerol stocks (20%, v/v) at −80 °C for long-term storage.

### 2.3 Morphological characteristics

The morphological characteristics of strain LZZY-S40 were investigated using both light microscopy and scanning electron microscopy (SEM; Axia ChemiSEM™, Thermo Fisher Scientific, USA) after cultivation on ISP medium 3 at 28 °C for 14 days. For scanning electron microscopy, samples were prepared according to the protocol described by He et al. (2015). Cultural characteristics, including colony morphology, the color of aerial and substrate mycelia, and the production of diffusible pigments, were recorded after a 14-day incubation on ISP media 2–6 at 28 °C. Color assessments were standardized using the ISCC-NBS color charts, to ensure consistent and reproducible descriptions (Shirling and Gottlieb, 1966).

### 2.4 Taxonomic identification based on phylogenetic and genomic analyses

#### 2.4.1 16S rRNA gene sequencing and phylogenetic analysis

For molecular identification, strain LZZY-S40 was cultured in GY liquid medium at 28 °C with shaking for 7 days. Cells were harvested by centrifugation, and genomic DNA was extracted according to the protocol described previously (Zhao et al., 2019). The nearly full-length 16S rRNA gene was amplified by PCR using the universal bacterial primers 27F (5′-AGAGTTTGATCCTGGCTCAG-3′) and 1541R (5′-AAGGAGGTGATCCAGCC-3′) under standard conditions (Springer et al., 1993). The PCR product was purified, ligated into the pMD19-T cloning vector (Takara, Osaka, Japan), and then sequenced commercially.

The resulting 16S rRNA gene sequence was assembled and compared with closely related sequences in the EzBioCloud database (https://www.ezbiocloud.net/) to determine sequence similarity. This sequence has been deposited in the GenBank database under accession number PX884312. A phylogenetic tree was constructed using the neighbor-joining method implemented in MEGA, and bootstrap support was calculated from 1,000 replicates.

#### 2.4.2 Genome sequencing and genome-based taxonomic analysis

Whole-genome sequencing of strain LZZY-S40 was conducted at the Beijing Genomics Institute (Shenzhen, China) using the DNBSEQ platform. Genomic DNA was randomly sheared to generate paired-end libraries. Raw reads of low quality were removed, and high-quality reads were *de novo* assembled using SPAdes version 3.15.5. Assembly quality was evaluated based on sequencing depth, N50 value, and GC content. Genome completeness was further assessed using BUSCO version 5.4.3 with the bacteria_odb10 dataset. The genome sequence has been deposited in GenBank under accession number JBTNTF000000000.

To determine the species-level taxonomic affiliation of strain LZZY-S40, average nucleotide identity (ANI) values between its genome and those of closely related *Streptomyces* species were calculated using the pyANI package with the BLAST-based ANIb algorithm under default parameters. Digital DNA–DNA hybridization (dDDH) values were estimated using the Genome-to-Genome Distance Calculator (GGDC) version 3.0 (https://ggdc.dsmz.de/) with formula 2, which is recommended for draft genomes.

For phylogenomic analysis, a genome-scale phylogenetic tree was inferred using PhyloPhlAn version 3.0. Universal single-copy core genes shared among the selected genomes were identified, aligned, and concatenated; a maximum-likelihood tree was then reconstructed using FastTree under default settings. Bootstrap support was assessed over 1,000 replicates, and the resulting tree was visualized using the Interactive Tree of Life (iTOL) platform (https://itol.embl.de/).

### 2.5 *In vitro* antifungal activity assay

The *in vitro* antifungal activity of strain LZZY-S40 was evaluated against nine phytopathogenic fungal species: *Exobasidium vexans, Gibberella zeae, Fusarium oxysporum, Pythium spinosum, Colletotrichum graminicola, Exserohilum turcicum, Rhizoctonia cerealis, Coniella diplodiella*, and *Pseudoperonospora cubensis*. All pathogenic fungi were obtained from Northeast Agricultural University (Harbin, China).

Antifungal activity was assessed using the dual-culture plate method on potato dextrose agar (PDA) as previously described (Zuo et al., 2025). Briefly, strain LZZY-S40 was point-inoculated at the margin of PDA plates and incubated at 28 °C for 3 days. Subsequently, a fresh mycelial plug of each target phytopathogen was placed at the opposite margin of the same plate. Control plates contained only the phytopathogen (Adra et al., 2024). All plates were incubated at 28 °C until the mycelia in the control plates fully colonized the medium. The inhibitory effect of strain LZZY-S40 on fungal growth was evaluated by measuring the inhibition zone formed between the strain LZZY-S40 and the pathogen. The percentage inhibition rate was calculated using the formula: Inhibition rate (%) = *R*_1_/*R* × 100%, where *R*_1_ represents the width of the inhibition zone and *R* represents the distance between the pathogen and the actinobacterium (Yu et al., 2020). All experiments were conducted in triplicate, and the mean values were used for subsequent analyses.

### 2.6 Wheat seedling growth promotion assay

The plant growth-promoting effect of strain LZZY-S40 was evaluated using a wheat seedling bioassay. Wheat seeds were surface-sterilized with 2% (v/v) sodium hypochlorite for 5 min and subsequently rinsed thoroughly with sterile distilled water. The sterilized seeds were then placed on sterile filter paper in Petri dishes.

Spore suspensions of strain LZZY-S40 were prepared at three concentrations (10^4^, 10^5^, and 10^6^ CFU ml^−1^). For each treatment, 30 seeds were used and distributed into three Petri dishes, with 10 seeds per dish. Seeds treated with sterile distilled water served as the negative control.

The seedlings were incubated in a growth chamber at 25 °C under a 16 h light/8 h dark photoperiod. After 7 days, all seedlings within each plate were measured, and the mean value of each plate was calculated. The final result for each treatment was determined by taking the mean of the 3 plates (*n* = 3). Measurements of total root length, number of root tips, shoot length, and fresh biomass were recorded.

### 2.7 Evaluation of antimicrobial-related traits

#### 2.7.1 Protease activity assay

Protease activity of strain LZZY-S40 was evaluated using an agar diffusion assay on skim milk agar. Briefly, actively growing cultures of strain LZZY-S40 were inoculated onto plates containing 1% (w/v) skim milk and incubated at 28 °C for 5 days. Protease activity was indicated by the formation of clear hydrolysis zones surrounding the inoculation sites. All assays were performed in triplicate to ensure reproducibility.

#### 2.7.2 Bioinformatic analysis of secondary metabolite biosynthetic gene clusters

The genome sequence of strain LZZY-S40 was analyzed to assess its potential for secondary metabolite biosynthesis through genome mining. Biosynthetic gene clusters (BGCs) were predicted and annotated using the antiSMASH platform (version 8.0.4; https://antismash.secondarymetabolites.org/) with default parameters (Medema et al., 2011). The analysis focused on identifying putative BGCs and comparing them with reference clusters in the Minimum Information about a Biosynthetic Gene Cluster (MIBiG) database.

#### 2.7.3 Fermentation, extraction, and LC-MS-based metabolomic analysis

Strain LZZY-S40 was cultivated on ISP 3 agar plates at 28 °C for 7 days, and spores were harvested for seed culture preparation. The spores were inoculated into 250 ml Erlenmeyer flasks containing 50 ml of seed medium (pH 7.2–7.4) and incubated at 28 °C for 48 h with shaking at 250 rpm. Subsequently, 8% (v/v) of the seed culture was transferred into fermentation medium and cultivated under the same conditions for 7 days. After fermentation, the culture broth was centrifuged at 12,000 rpm for 10 min to separate the supernatant from the mycelial biomass. The supernatant was extracted three times with ethyl acetate, while the mycelial pellet was extracted with methanol. The organic phases were concentrated under reduced pressure at temperatures below 40 °C, and the resulting crude extracts were combined for metabolomic analysis.

Untargeted metabolomic profiling was performed using liquid chromatography-mass spectrometry (LC-MS) on an AB SCIEX Triple TOF 6600 system equipped with an electrospray ionization (ESI) source. Data were acquired in both positive and negative ionization modes across an m/z range of 50–1200. Chromatographic separation was achieved using a gradient elution program at a flow rate of 400 μl min^−1^, with the column temperature maintained at 40 °C.

Raw data were converted to mzML format and subsequently processed using a standardized metabolomics workflow, including peak detection, retention time alignment, and signal normalization. Metabolite annotation was performed by matching MS/MS spectra against public databases including METLIN, HMDB, MassBank, and MoNA. Features with low confidence scores or those detected in blank control samples were excluded before downstream analysis.

### 2.8 Evaluation of plant growth-promoting traits

#### 2.8.1 Detection of indole-3-acetic acid production

For the detection of indole-3-acetic acid (IAA), spores of strain LZZY-S40 were initially inoculated into seed medium containing 0.4% yeast extract, 1% malt extract, 0.4% glucose, and 0.4% CaCO_3_ (pH 7.2–7.4), and incubated at 28 °C with shaking at 250 rpm for 2 days. Subsequently, a 4% (v/v) inoculum was transferred into fermentation medium composed of 4% soluble starch, 2.5% soybean meal powder, 2.5% peptone, 1% glucose, 0.2% CaCO_3_, 0.8% MgSO_4_·7H_2_O, 0.6% FeSO_4_·7H_2_O, 0.2% ZnSO_4_·7H_2_O, 0.2% MnSO_4_·H_2_O, 0.05% CoCl_2_·6H_2_O, and 0.2% Na_2_MoO_4_·2H_2_O (pH 7.0), and cultured under the same conditions for 10 days.

After incubation, the culture broth was centrifuged to obtain the cell-free supernatant. Qualitative detection of IAA was carried out using the Salkowski colorimetric method (Fierros et al., 2024). 1 ml of culture supernatant was mixed with 2 ml of Salkowski reagent. The development of a pink coloration indicated the presence of IAA production.

#### 2.8.2 Siderophore production assay

Siderophore production by strain LZZY-S40 was assessed using chrome azurol S (CAS) agar. The strain was spot-inoculated onto CAS agar plates and incubated at 28 °C for 5 days. The formation of orange halos surrounding the colonies was considered indicative of siderophore secretion.

#### 2.8.3 Qualitative assessment of nitrogen fixation and ACC deaminase activity

The nitrogen fixation potential of strain LZZY-S40 was preliminarily evaluated based on its ability to grow on Ashby nitrogen-free solid medium. The strain was inoculated onto Ashby agar plates and incubated at 28 °C for 7 days. Visible colony growth was interpreted as an indication of potential nitrogen-fixing capability.

ACC deaminase activity was qualitatively assessed using DF agar supplemented with ACC (ADF medium), following the method described previously (Jin et al., 2024). Growth on ADF medium was considered preliminary evidence of ACC deaminase activity.

### 2.9 Statistical analysis

All experiments were performed with at least three independent biological replicates unless otherwise stated. Data were presented as the mean ± standard deviation (SD). The Student's *t*-test (two-tailed) was used to assess statistical significance. In all graphical representations, error bars represent the standard deviation (SD) of biological replicates. Statistical significance was indicated as ^*^P < 0.05, ^**^P < 0.01, and ^***^P < 0.001.

## 3 Results

### 3.1 Taxonomic identification of *Streptomyces* sp. LZZY-S40

*Streptomyces* sp. LZZY-S40 was isolated from the rhizosphere soil of tea plants. To determine its taxonomic position, a comprehensive analysis was conducted based on morphological characteristics, phylogenetic relationships, and genome-level features.

The growth characteristics of strain LZZY-S40 varied across different ISP media (ISP2–ISP6), with optimal growth observed on ISP3. On this medium, the strain produced well-developed light-gray aerial mycelia and grayish-red substrate mycelia, along with a dark, vivid pink soluble pigment. Robust growth was also observed on ISP4, where white aerial mycelia and moderately pink substrate mycelia formed, accompanied by a vivid pink diffusible pigment. In contrast, growth on the remaining media (ISP2, ISP5, and ISP6) was limited, characterized by the absence of aerial mycelia, the presence of light-yellow substrate mycelia, and no detectable soluble pigment production. The detailed cultural characteristics of the strain are summarized in Supplementary Table 1.

**Figure 1:**
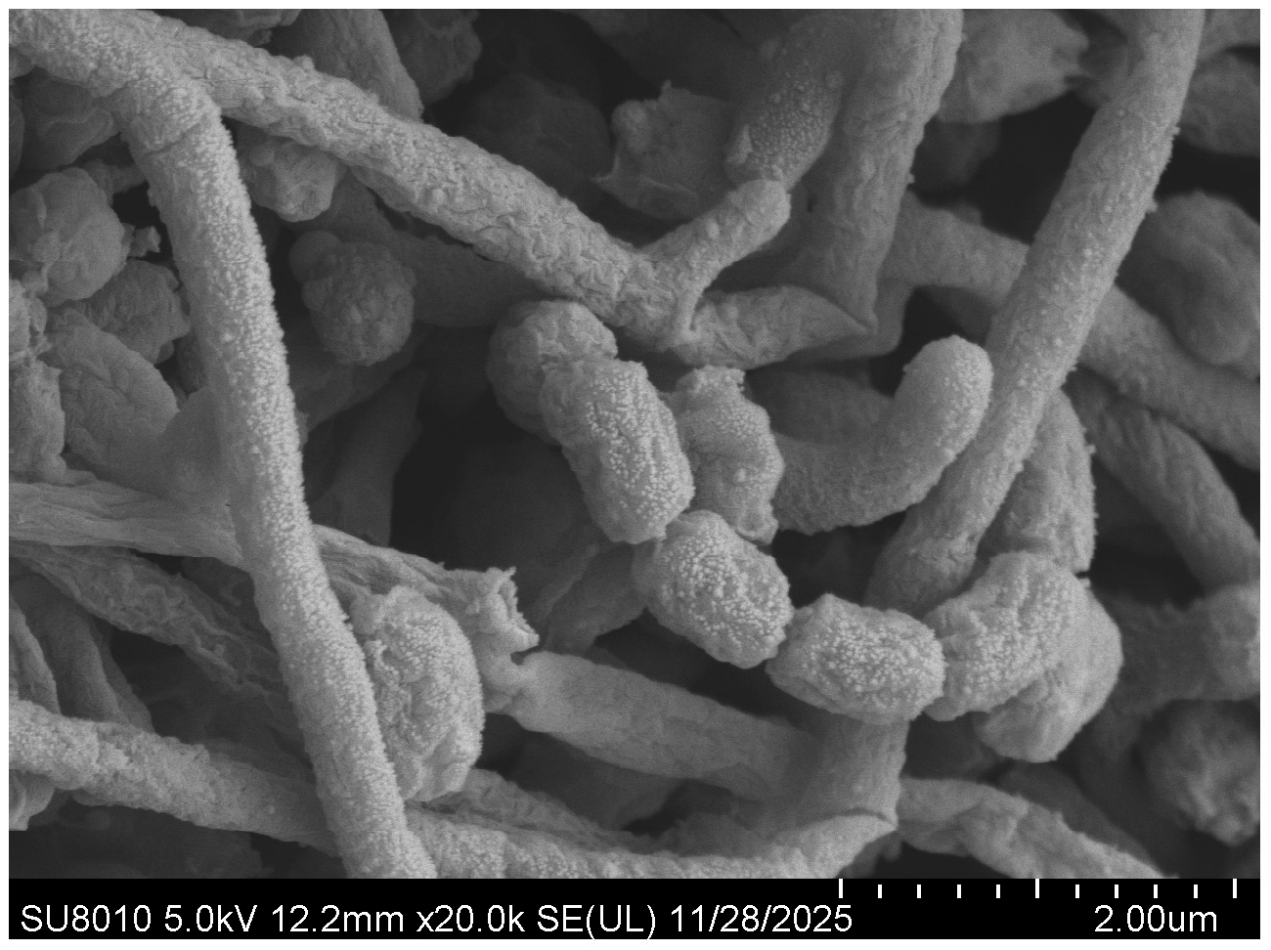
Scanning electron micrograph of strain LZZY-S40 grown on ISP medium 3 for 2 weeks at 28 °C.

Scanning electron microscopy (SEM) revealed that strain LZZY-S40 developed well-branched substrate hyphae and abundant aerial mycelia, which differentiated into straight or spiral spore chains. These chains were composed of cylindrical spores measuring approximately 0.45–0.85 μm in width and 0.65–1.12 μm in length, with a rough surface texture (Figure 1). These morphological traits were consistent with those typical of the genus *Streptomyces*.

**Figure 2:**
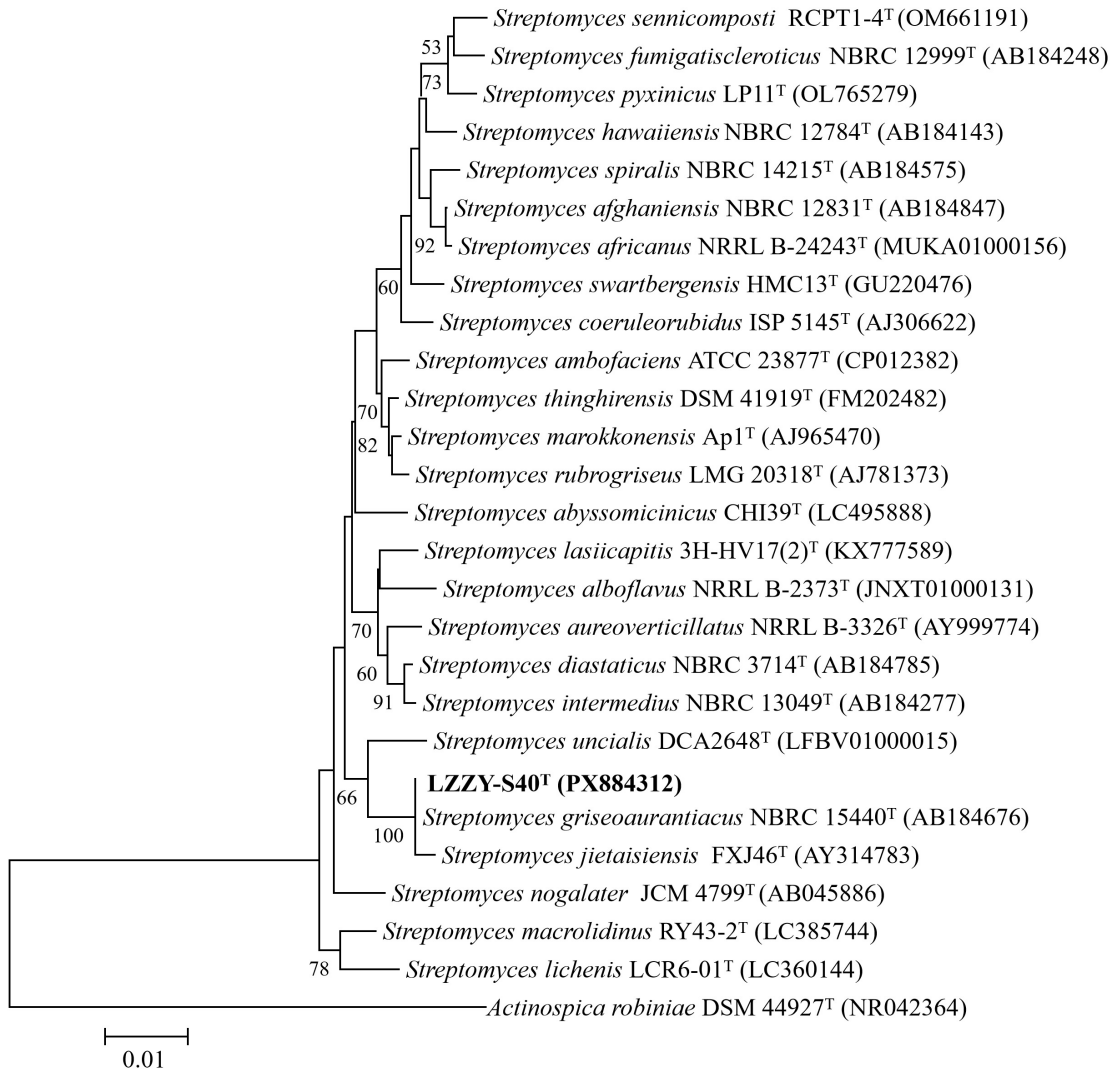
Neighbor-joining tree based on 16S rRNA gene sequences showing the relationship between strain LZZY-S40 and related taxa. Numbers at nodes are bootstrap values (percentages of 1,000 replications); only values >50% are shown. *Actinospica robiniae* DSM 44927^T^ (NR042364) was used as an outgroup. Bar, 0.010 nucleotide substitutions per site.

Phylogenetic analysis based on the 16S rRNA gene sequence further clarified the taxonomic affiliation of strain LZZY-S40. In the neighbor-joining phylogenetic tree, it clustered closely with *Streptomyces griseoaurantiacus* and *Streptomyces jietaisiensis*, supported by a bootstrap value of 100% (Figure 2). BLAST analysis against the EzBioCloud database indicated 100% 16S rRNA gene sequence identity with *S. griseoaurantiacus* NBRC 15440 and 99.7% identity with *S. jietaisiensis*.

To confirm species-level classification, genome-based comparative analyses were performed. The average nucleotide identity (ANI) between strain LZZY-S40 and *S. griseoaurantiacus* was 99.10%, exceeding the widely accepted species delineation threshold of 95–96%. Similarly, the digital DNA–DNA hybridization (dDDH) value was 92.5%, substantially higher than the 70% threshold for species demarcation. Furthermore, a genome-scale phylogenetic tree constructed from concatenated conserved core genes placed strain LZZY-S40 within the same clade as *S. griseoaurantiacus* NBRC 15440 (Supplementary Figure S1), further supporting their close evolutionary relationship. Collectively, these results indicated that strain LZZY-S40 should be classified as *Streptomyces griseoaurantiacus*.

### 3.2 Antifungal activity of strain LZZY-S40 against phytopathogenic fungi

**Figure 3:**
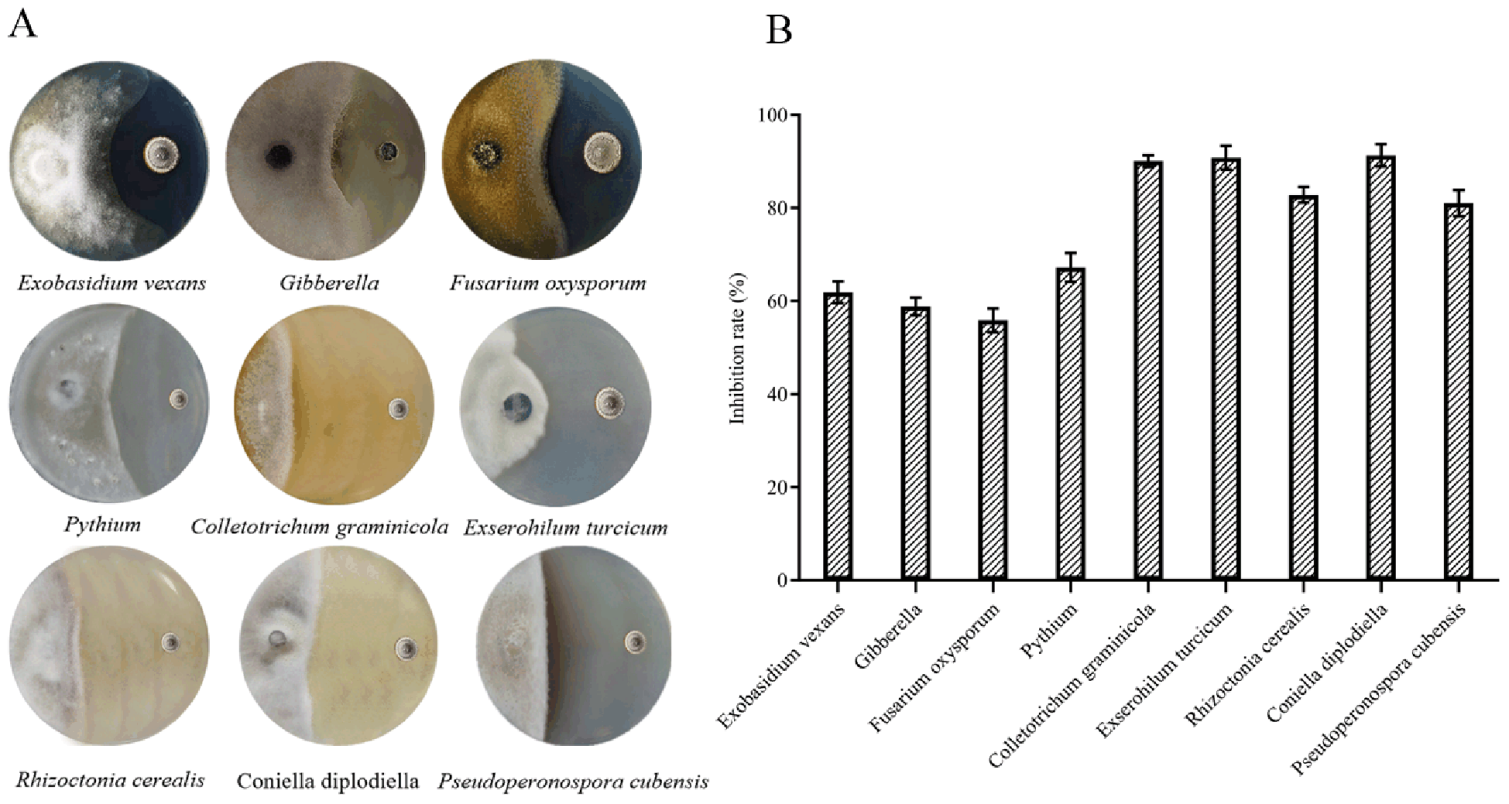
Antifungal activity of strain LZZY-S40 against the tested fungi. **(A)** Dual culture plate assay against tested fungi; **(B)** Inhibition rate against the tested fungi. Error bars show standard deviations. Data are presented as mean ± SD of three biological replicates.

The antifungal activity of strain LZZY-S40 was assessed using a dual-culture plate assay against a diverse panel of phytopathogenic fungi. Strain LZZY-S40 exhibited broad-spectrum antagonistic activity against all 9 tested pathogens, including *Exobasidium vexans, Gibberella zeae, Fusarium oxysporum, Pythium spinosum, Colletotrichum graminicola, Exserohilum turcicum, Rhizoctonia cerealis, Coniella diplodiella*, and *Pseudoperonospora cubensis*. Pronounced inhibition of mycelial growth was observed in the confrontation assays, and quantitative analysis revealed inhibition rates ranging from 55.8% to 92.8%, based on mean values from three independent biological replicates (Figure 3). Among the tested fungi, *Exserohilum turcicum* was the most sensitive to strain LZZY-S40, with the highest mean inhibition rate reaching 92.8%. These results revealed that strain LZZY-S40 possessed strong antagonistic potential and indicated its potential as a biocontrol agent against a wide range of plant-pathogenic fungi.

### 3.3 Plant growth-promoting effects of strain LZZY-S40

**Figure 4:**
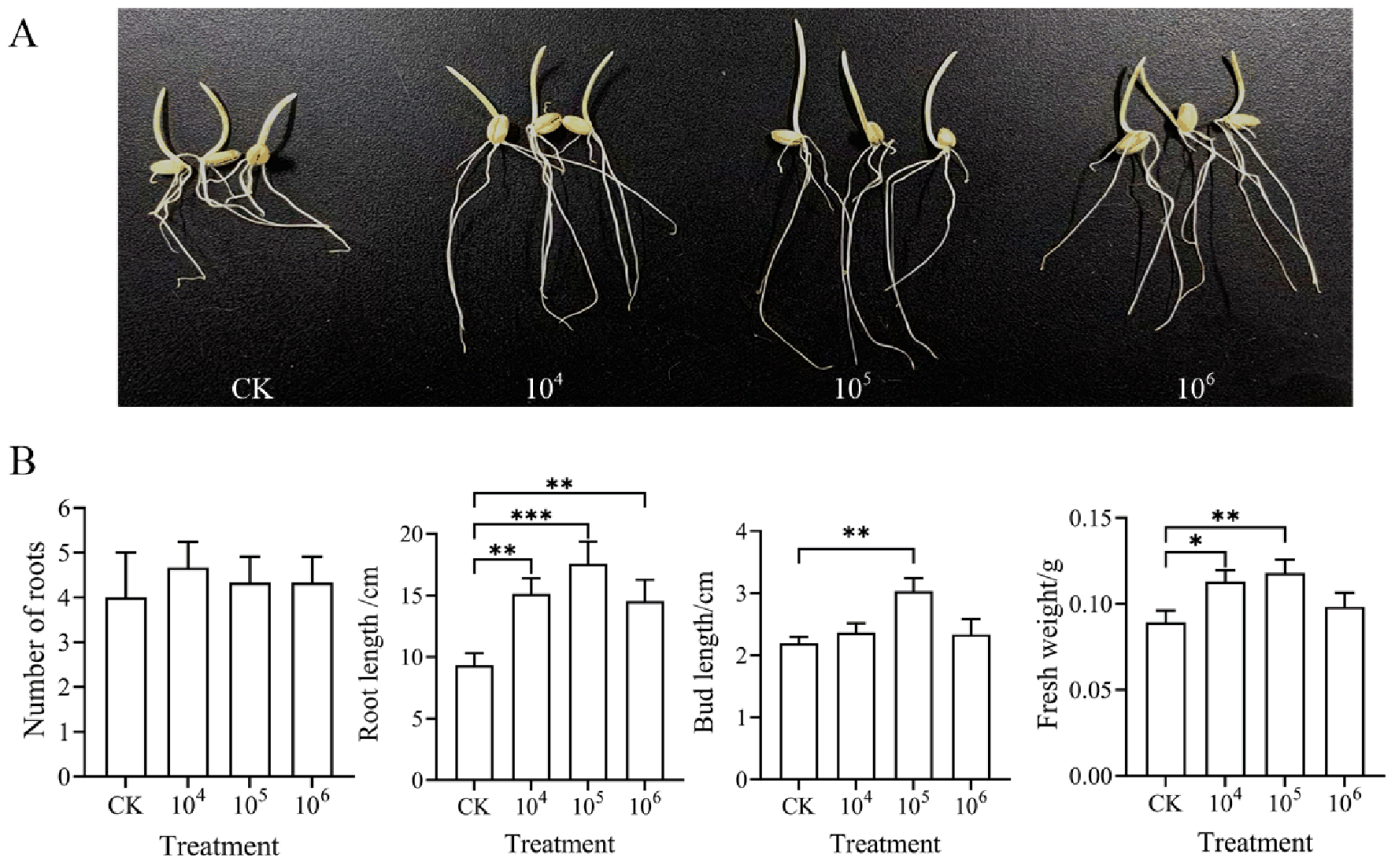
Growth response of wheat seedlings to different concentrations of the spore suspensions of *Streptomyces* sp. LZZY-S40. **(A)** Phenotypic observation of wheat seedlings under sterile water and spore suspension treatments at 10^4^, 10^5^, and 10^6^ CFU·ml^−1^. Three seedlings shown for each treatment in the figure represent one randomly selected seedling from each of the three Petri dishes, which serve as a representative sample of the full set of data from the respective treatment. **(B)** Recorded measurements of root number, root length, bud length, and fresh weight under the same treatments mentioned above. Error bars show standard deviations. Data are presented as mean ± SD of three biological replicates. *P*-values were determined by Student's *t*-test. *P < 0.05, **P < 0.01, and ***P < 0.001.

The plant growth-promoting properties of strain LZZY-S40 were evaluated by inoculating wheat seeds with spore suspensions at three concentrations (104, 105, and 106 CFU·ml^−*l*^). Seedling parameters—including number of root tips, root length, bud length, and fresh weight-were measured (Figure 4A). No significant differences were observed in root tip number among the treatments. However, inoculation with spore suspensions of strain LZZY-S40 significantly promoted root elongation, bud development, and seedling biomass accumulation, with the most pronounced effects observed at a concentration of 10^5^ CFU·mL^−1^. At this concentration, root length, bud length, and fresh weight increased by 88.3%, 37.9%, and 32.1%, respectively, compared with the control, compared to the control (Figure 4B). These results indicated that the ability of strain LZZY-S40 to promote early seedling growth and biomass production, suggesting its potential for application in sustainable agricultural practices.

### 3.4 Antimicrobial-related traits and secondary metabolite profiling of strain LZZY-S40

#### 3.4.1 Extracellular protease activity of strain LZZY-S40

The extracellular protease activity of strain LZZY-S40 was qualitatively evaluated using a plate-based assay. Clear hydrolysis zones were observed around the colonies of strain LZZY-S40 (Supplementary Figure S2), indicating its ability to degrade proteinaceous substrates and suggesting the production of extracellular proteolytic enzymes. Given that proteases can contribute to the degradation of fungal cell wall–associated proteins, this enzymatic activity may represent a potential factor contributing to the antifungal activity of strain LZZY-S40.

#### 3.4.2 Genome-based analysis of secondary metabolite biosynthetic potential

**Figure 5:**
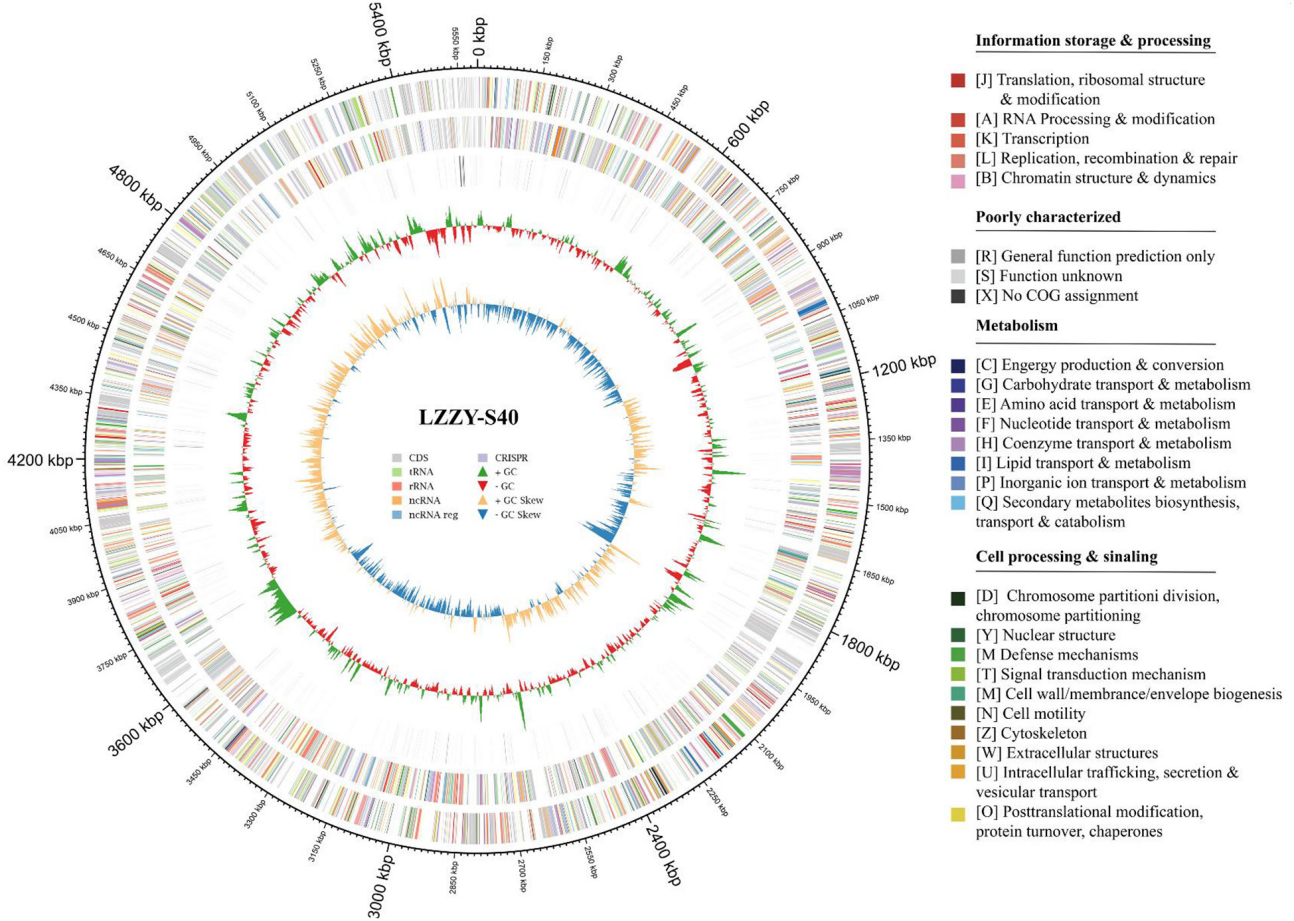
Circular genome map of strain LZZY-S40. The outermost layer displays the genomic coordinate scale in kilobase pairs (kbp). Moving inward from the outer edge, concentric rings represent: genome coordinates indicated by black tick marks; coding sequences (CDS), transfer RNA (tRNA), and other genetic elements, color-coded according to functional category; GC content shown as a blue line; and GC skew depicted as a green line. The colored bar on the right illustrates the clusters of orthologous groups (COG) functional classification, with colors representing major functional categories: information storage and processing, metabolism, cellular processes and signaling, and poorly characterized or unknown functions.

The draft genome of strain LZZY-S40 was sequenced to further explore its biosynthetic potential. The genome size is 7.4 Mbp with a GC content of 72.94%, and the sequence has been deposited in GenBank under accession number JBTNTF000000000. Genome annotation revealed 7,728 predicted protein-coding genes. In addition, the genome contains 73 tRNA genes and five rRNA genes, including three copies of 5S rRNA and one copy each of 16S and 23S rRNA, as well as 81 small RNA (sRNA) genes (Figure 5).

To assess the secondary metabolic potential of strain LZZY-S40, genome analysis was performed using antiSMASH, which identified 29 putative biosynthetic gene clusters (BGCs) (Table 1). These include gene clusters associated with polyketide synthases (PKSs), non-ribosomal peptide synthetases (NRPSs), terpenes, siderophores, and other secondary metabolites such as ectoine, melanin, and nucleoside. These findings indicate that strain LZZY-S40 harbors a diverse repertoire of secondary metabolite biosynthetic pathways, a characteristic feature of metabolically versatile *Streptomyces* species.

**Table 1:** Major secondary metabolite biosynthetic gene clusters identified in strain LZZY-S40 using AntiSMASH v8.0.4.

Cluster No.	BGC type	BGC length (bp)	Most similar known cluster (%)
1	Nucleoside	20,836	Cinnamycin B (38%)
2	Melanin	10,611	Melanin (83%)
3	NRPS	75,654	Desferrioxamine E (58%)
4	T3PKS, guanidinotides	41,088	Ketomemicin B3, ketomemicin B4 (45%)
5	Azole-containing-RiPP	24,874	(2R,3S,4S)-5-fluoro-2,3,4-trihydroxypentanoic acid (50%)
6	Terpene	32,117	Isorenieratene (72%)
7	T2PKS	72,516	Prunipeptin (78%)
8	Terpene	21,041	Avermitilol (83%)
9	Aminopolycarboxylic-acid	13,460	Prunipeptin (78%)
10	Terpene, NRPS-like, T1PKS	58,969	6-methylsalicyclic acid (99%)
11	NRPS	53,547	Pyreudione A/B/C/D/E (74%)
12	Terpene-precursor	21,014	(2R,3S,4S)-5-fluoro-2,3,4-trihydroxypentanoic acid (40%)
13	Terpene-precursor	21,044	Sesterfisherol/sesterfisheric acid (43%)
14	Terpene	21,086	Albaflavenone (85%)
15	Terpene-precursor	21,137	Astallatene (46%)
16	Prodigiosin, NRPS-like, T1PKS	54,736	Undecylprodigiosin (94%)
17	T1PKS	47,560	Asperlactone (66%)
18	Terpene	22,151	Geosmin (99%)
19	RiPP-like	11,302	14-hydroxyisochainin (27%)
20	T1PKS	45,490	Asperlactone (66%)
21	Nl-siderophore	30,061	Speibonoxamine/desoxy-desferrioxamine D1/desferrioxamine B (51%)
22	NRPS-like	23,034	Gamexpeptide C (63%)
23	Furan	21,110	Asukamycin (60%)
24	RiPP-like	10,216	Informatipeptin (56%)
25	Ectoine	10,402	Ectoine (85%)
26	Terpene	25,806	Atolypene A/atolypene B (51%)
27	Terpene	18,674	Hopene (70%)
28	NRPS	26,399	Bovienimide A (71%)
29	NRPS	5,596	Bicornutin A1/bicornutin A2 (70%)

Notably, several BGCs showed high similarity to gene clusters responsible for the biosynthesis of known bioactive compounds. One BGC exhibited significant similarity (94%) to the undecylprodigiosin biosynthetic gene cluster from *Streptomyces coelicolor* A3(2), which is associated with the production of undecylprodigiosin, a red-pigmented pyrrole antibiotic with well-documented antifungal activity. This high level of conservation suggests that strain LZZY-S40 may have the potential to produce undecylprodigiosin or structurally related prodigiosin-family compounds with antimicrobial properties.

Another BGC of particular interest (BGC 23) was predicted to encode enzymes potentially related to the biosynthesis of a compound containing an epoxide group, rather than the previously hypothesized furan structure. This BGC belongs to a family of oxidoreductases and shows strong similarity to the asukamycin biosynthetic gene cluster, which is known to mediate the biosynthesis of epoxyquinone polyenes. Additional support for this functional prediction comes from a three-gene subcluster predicted to synthesize 2-amino-3-hydroxycyclopent-2-enone, a conserved structural moiety shared by both asukamycin and manumycin. Although no direct homolog of the manumycin biosynthetic gene cluster was detected, the observed similarity to the asukamycin cluster and the presence of manumycin-related biosynthetic motifs suggest that this BGC may be associated with the biosynthetic potential of epoxyquinone polyene compounds such as manumycin A, which has been reported to exhibit antifungal and insecticidal activities.

In addition, the genome harbors several BGCs linked to melanin biosynthesis, siderophore biosynthesis, and terpene biosynthesis, including clusters predicted to encode isorenieratene, avermitilol, vermitilol, albaflavenone, geosmin and hopene. The remaining BGCs exhibit low similarity to known entries in the MIBiG database, indicating the potential existence of novel or poorly characterized secondary metabolite pathways in strain LZZY-S40.

#### 3.4.3 Metabolomic analysis of secondary metabolites in strain LZZY-S40

Metabolomic analysis of the fermentation broth of strain LZZY-S40 was conducted using LC-MS. Initially, 3,300 metabolites were detected. After filtering out compounds present in the control group and those with low annotation confidence, a total of 598 metabolites were retained for further analysis (Supplementary Table S2).

Functional annotation suggested that these metabolites were associated with diverse biological activities, such as cytotoxicity, toxin-related functions, chemical sensitization, and herbicidal or insecticidal effects, indicating the broad secondary metabolic potential of strain LZZY-S40.

Notably, several metabolites that have previously been reported to exhibit antifungal activity were tentatively annotated based on LC–MS data, including undecylprodigiosin, manumycin A, bipolamide B, tryptophol, and 2-hexyl-5-methylresorcinol (Table 2). These compounds have been reported to exhibit inhibitory effects against fungal pathogens in previous studies and may potentially contribute to the antifungal activity of strain LZZY-S40 observed in plate confrontation assays.

**Table 2:** Tentatively annotated antifungal-related metabolites in the fermentation extract of *Streptomyces* sp. LZZY-S40 based on LC-MS analysis.

Name	Formula	Calc. MW	Description	Bioactivity
Undecylprodigiosin	C_25_H_35_N_3_O	393.27805	Tripyrrole	Antibacterial, antifungal, antimalarial, cytotoxic activity
Manumycin A	C_31_H_38_N_2_O_7_	550.26757	Polyketide	Cytotoxic activity, antifungal, antimicrobial, insecticidal
Bipolamide B	C_12_H_19_NO	193.14658	Triene fatty acid amide	Antifungal
Tryptophol	C_10_H_11_NO	161.08423	Tryptophan metabolite	Antibacterial, autoantibiotic, antifungal, phytotoxic
2-hexyl-5-methylresorcinol	C_13_H_20_O_2_	208.14645	Dialkylresorcinol	Antibacterial, antifungal

### 3.5 Plant growth-promoting traits associated with strain LZZY-S40

To further elucidate the mechanisms underlying the plant growth-promoting effects of strain LZZY-S40, several functional traits associated with plant growth promotion were examined. Qualitative assays suggested that strain LZZY-S40 produced indole-3-acetic acid (IAA), as indicated by the development of a characteristic pink color following the addition of Salkowski reagent (Supplementary Figure 3A). Additionally, siderophore production was verified by the formation of distinct orange halos on chrome azurol S (CAS) agar plates (Supplementary Figure 3B), suggesting its potential to enhance iron acquisition and bioavailability for plant growth.

Beyond these direct growth-promoting capabilities, strain LZZY-S40 also displayed traits linked to nutrient mobilization and stress mitigation. The strain grew on Ashby nitrogen-free solid medium after 7 days of incubation at 28 °C, indicating a potential capacity for nitrogen fixation (Supplementary Figure 3C). Moreover, its ability to utilize 1-aminocyclopropane-1-carboxylate (ACC) as a sole nitrogen source on DF agar (ADF medium) demonstrated ACC deaminase (ACCD) activity (Supplementary Figure 3D), which may help lower ethylene levels in plants under stress, thereby promoting root elongation and seedling establishment.

Collectively, these multiple plant growth-promoting traits provide functional evidence supporting the growth-enhancing potential of strain LZZY-S40 and offer mechanistic insights into its beneficial interactions with plants.

## 4 Discussion

With increasing pressure to reduce reliance on chemical pesticides, *Streptomyces*-based biocontrol agents have emerged as promising alternatives in sustainable agriculture (da Silva et al., 2025). However, despite the growing number of reported antagonistic and plant growth-promoting strains, most studies remain largely phenotype-oriented and provide limited integrative evidence linking physiological traits with genomic potential and metabolic outputs (Zhou et al., 2025; Li L. et al., 2025). In particular, the mechanistic basis underlying the coexistence of antifungal and plant growth-promoting functions in individual *Streptomyces* strains remains insufficiently explored. In this study, *Streptomyces* sp. LZZY-S40 was investigated through an integrated framework combining phenotypic assays, enzymatic characterization, genome mining, and metabolomic profiling, enabling a multi-layered interpretation of its functional traits.

The broad antifungal spectrum observed for strain LZZY-S40 suggests that its antagonistic capacity is unlikely to rely on a single inhibitory factor but may instead involve multiple complementary mechanisms. Such multifunctional inhibition strategies are frequently reported in *Streptomyces* species and are considered advantageous for suppressing diverse phytopathogens in complex soil environments (Alam et al., 2022). To elucidate the basis of this phenotype, enzymatic assays, genome mining, and metabolomic analyses were integrated to construct a multidimensional interpretation of its antagonistic traits. Protease-mediated disruption of fungal structural integrity has been widely reported in actinomycetes and is considered an important factor facilitating fungal inhibition and enhancing the efficacy of antimicrobial metabolites (Kumari and Rao, 2025). Extracellular protease production, as demonstrated by qualitative assays, suggests that strain LZZY-S40 may degrade proteinaceous components of fungal cell walls and membranes. In this context, proteolytic activity in strain LZZY-S40 may represent an initial or synergistic layer of antagonism, contributing to fungal susceptibility during microbial interactions.

Beyond enzymatic activity, secondary metabolites are widely recognized as major contributors to the antagonistic activities of *Streptomyces* species. In recent years, advances in genome sequencing have revealed that *Streptomyces* genomes typically possess large genome sizes, high GC content, and an exceptional abundance of biosynthetic gene clusters, which collectively underpin their remarkable capacity to produce structurally diverse bioactive compounds (Lodi et al., 2025). Consistent with these genomic characteristics, genome mining of strain LZZY-S40 revealed the presence of 29 putative biosynthetic gene clusters, highlighting its considerable potential for secondary metabolite production. Among these, gene clusters putatively associated with the biosynthesis of undecylprodigiosin and manumycin A were identified. These compounds have been reported to exhibit antifungal activity through distinct mechanisms, including membrane perturbation, interference with cellular redox homeostasis, and inhibition of key metabolic or signaling pathways (Cho et al., 2015; Chatragadda et al., 2021; Lee et al., 2024; Wang et al., 2025). Consistent with genomic predictions, LC-MS-based metabolomic profiling revealed a complex secondary metabolite landscape in the fermentation extracts of strain LZZY-S40. Several compounds tentatively annotated as undecylprodigiosin and manumycin A were detected based on LC–MS spectral matching, providing congruent evidence linking genomic potential with metabolic expression. In addition, several other bioactive metabolites, including bipolamide B, tryptophol, and 2-hexyl-5-methylresorcinol, were also tentatively annotated. These metabolites have been reported to modulate fungal growth, morphogenesis, membrane integrity, or signaling processes (Auddy et al., 2022; Liu et al., 2024). The presence of multiple putative antifungal metabolites suggests that strain LZZY-S40 may employ a chemically diverse arsenal of secondary metabolites to inhibit competing microorganisms. Such metabolite diversity is a common ecological strategy among *Streptomyces* species, enabling them to compete effectively within highly competitive soil and rhizosphere microbiomes.

Nevertheless, several candidate metabolites were tentatively annotated through LC–MS analysis, their identities and specific roles in antifungal activity require further verification. Future studies involving comparison with authentic standards, compound isolation and purification, structural characterization, as well as functional validation of the corresponding biosynthetic gene clusters will be necessary to confirm their production and clarify their contributions to the antifungal phenotype of strain LZZY-S40. Taken together, these findings suggest that the antifungal activity of strain LZZY-S40 may partially result from the combined effects of extracellular enzymes and a structurally diverse array of secondary metabolites, rather than from a single dominant compound. Such synergistic mechanisms may enhance the ecological competitiveness of the strain and reduce the likelihood of resistance development in target pathogens.

Beyond its antagonistic traits, strain LZZY-S40 also possesses several characteristics commonly associated with plant growth-promoting microorganisms. The production of indole-3-acetic acid (IAA) suggests a role in modulating root architecture and stimulating plant development, as this phytohormone plays a central role in regulating cell elongation and root system formation (Myo et al., 2019). Siderophore production may contribute to plant growth by improving iron availability in the rhizosphere, thereby alleviating iron limitation and enhancing nutrient uptake (Saini et al., 2026). In addition to enhancing plant nutrition, siderophore production may also suppress certain phytopathogens through iron competition, an ecological mechanism frequently observed in plant-associated beneficial microbes. The presence of ACC deaminase activity implies a potential role in mitigating ethylene-induced growth inhibition under stress conditions, while nitrogenase activity indicates a possible contribution to nitrogen acquisition in nutrient-limited environments (Ruanpanun and Nimnoi, 2020; Abderrahim et al., 2021; Du et al., 2022). Collectively, these traits point to a multifaceted plant growth-promoting strategy integrating hormonal modulation, nutrient mobilization, and stress mitigation, consistent with functional patterns observed in effective plant-associated *Streptomyces* strains.

From an ecological perspective, the coexistence of antifungal and plant growth-promoting traits may provide important advantages in rhizosphere environments, where microorganisms simultaneously compete with pathogens and interact with host plants (Su et al., 2023). The ability to produce antimicrobial metabolites while enhancing plant nutrient acquisition and stress tolerance may facilitate the establishment of beneficial plant–microbe associations and contribute to the suppression of soil-borne diseases (Li Z. et al., 2025; Quan et al., 2025).

To further substantiate the agricultural potential of strain LZZY-S40, comprehensive evaluations under both controlled greenhouse and field conditions are essential. Future research should focus on pot experiments to assess the strain's plant growth-promoting effects and its ability to suppress a broad spectrum of plant pathogens. Additionally, rhizosphere colonization assays are crucial for determining the strain's persistence and its interactions with the root environment. Pathogen challenge studies will be instrumental in evaluating the strain's *in vivo* biocontrol efficacy. Ultimately, large-scale field trials across diverse agricultural systems and soil types are required to establish the long-term effectiveness and sustainability of LZZY-S40 as a biofertilizer or biocontrol agent under realistic field conditions.

## 5 Conclusion

In this study, *Streptomyces* sp. LZZY-S40 was taxonomically identified based on phylogenetic and genomic analyses and systematically characterized for its antifungal and plant growth-promoting functions. The strain exhibited broad-spectrum antifungal activity against nine phytopathogenic fungi, with inhibition rates ranging from 55.8% to 92.8%, the strongest inhibition (92.8%) was observed against *Exserohilum turcicum*.

Mechanistic investigations suggested that LZZY-S40 produces extracellular proteases and harbors multiple biosynthetic gene clusters potentially associated with antimicrobial secondary metabolism, including gene clusters related to undecylprodigiosin and manumycin A biosynthesis. Consistently, LC-MS analysis putatively identified these compounds together with other bioactive metabolites, indicating that its antifungal activity of strain LZZY-S40 may be associated with the combined effects of extracellular enzymatic activity and chemically diverse secondary metabolites.

Concurrently, LZZY-S40 significantly enhanced early-stage wheat seedling development, increasing root length by 88.3%, shoot length by 37.9%, and fresh weight by 32.1%. This growth-promoting phenotype was supported by the detection of indole-3-acetic acid production, siderophore secretion, and enzymatic activities related to ACC deaminase and nitrogenase, suggesting coordinated functions in hormone regulation, nutrient acquisition, and stress mitigation.

Overall, this study demonstrates that *Streptomyces* sp. LZZY-S40 integrates strong antifungal capacity with pronounced plant growth-promoting traits. These findings provide a mechanistic framework for understanding its dual functional phenotype and support its potential application as a microbial resource for sustainable biological control and agricultural production systems.

## Data Availability

The original contributions presented in the study are publicly available. This data can be found here: MetaboLights, accession MTBLS14181.
