# Supplementary material for: Functional characterization and mechanistic basis of antifungal and plant growth-promoting traits of Streptomyces sp. LZZY-S40
Source: Front Microbiol. 2026 Apr 15;17:1800596. doi: 10.3389/fmicb.2026.1800596 (PMC13127372; doi:10.3389/fmicb.2026.1800596)
Supplement: Supplementary file 1 [file Supplementary_file_1.docx]

**Supplementary materials**

Functional characterization and mechanistic basis of antifungal and plant growth-promoting traits of *Streptomyces* sp. LZZY-S40

Lan Ye ^1^, Pinjiao Jin ^2^, Zhenhua Liu ^1^, Dongmiao Qin ^1^, Haolin Wang ^1^, Liyang Chu ^3*^

^1^School of Environmental and Food Engineering, Liuzhou Polytechnic University, Liuzhou, China

^2^Heilongjiang Academy of Black Soil Conservation & Utilization, Postdoctoral Station of Heilongjiang Academy of Agricultural Sciences, Harbin, China

^3^Yantai Key Laboratory of Characteristic Agricultural Bioresource Conservation & Germplasm Innovative Utilization, College of Life Sciences, Yantai University, Yantai, China

***Authors for correspondence:**

Liyang Chu, Yantai Key Laboratory of Characteristic Agricultural Bioresource Conservation & Germplasm Innovative Utilization, College of Life Sciences, Yantai University, 1 Siping Road, Yantai, 264005, PR China, E-mail: chuliyang@ytu.edu.cn

Running title: Functional and mechanistic basis of bioactivity in *Streptomyces* sp. LZZY-S40

**Supplementary** **Table S1:** Growth and cultural characteristics of strain LZZY-S40.

| **Medium** | **Growth** | **Aerial mycelium** | **Substrate mycelium** | **Diffusible pigment** |
| --- | --- | --- | --- | --- |
| ISP2 | Poor | None | Light yellow | None |
| ISP3 | Good | Light gray | Grayish red | Dark vivid pink |
| ISP4 | Moderate | White | Moderate pink | vivid pink |
| ISP5 | Poor | None | Light yellow | None |
| ISP6 | Poor | None | Pale yellow | None |

**
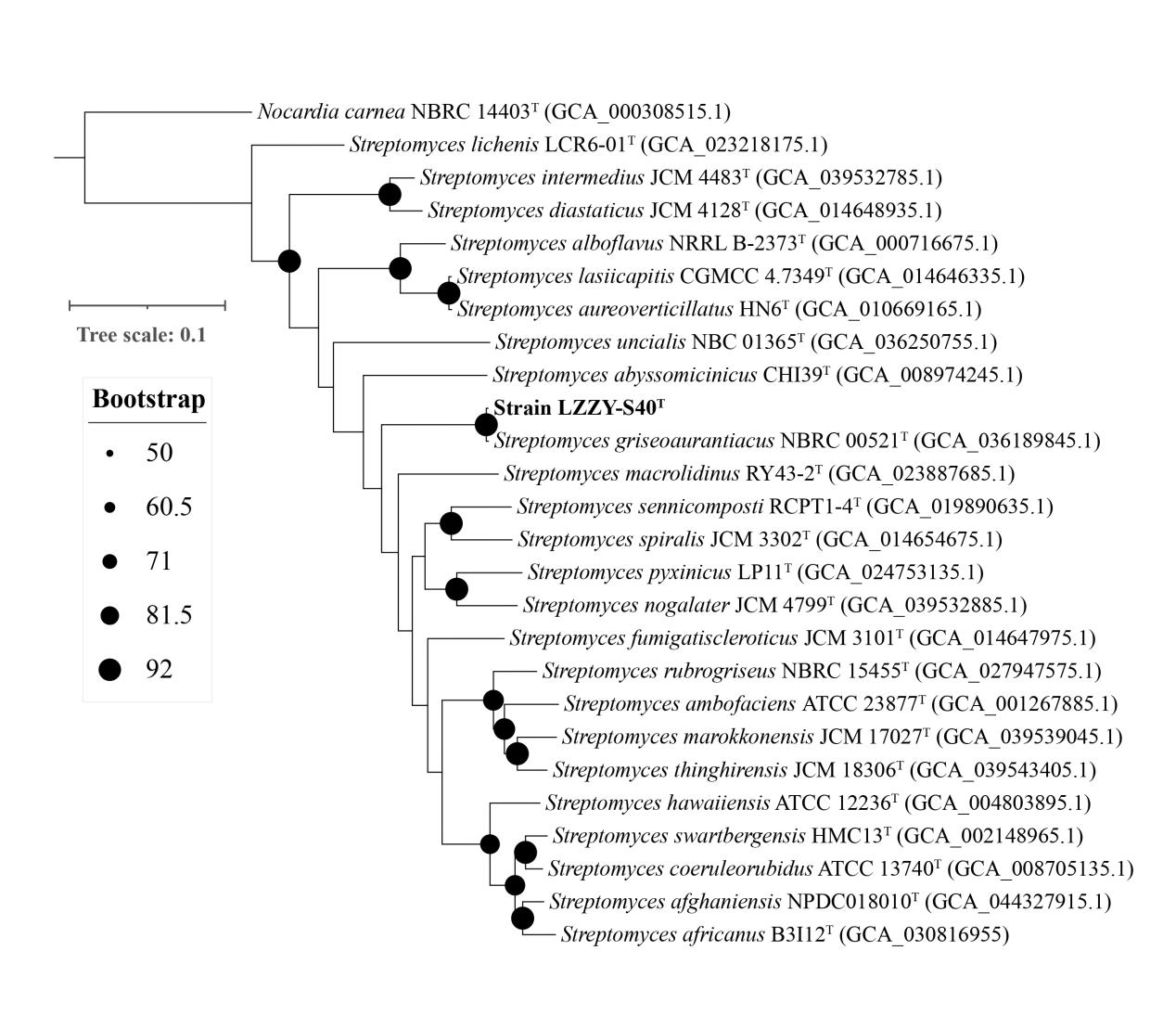
**

**Supplementary** **Figure S1** Phylogenomic tree based on 25 bacterial single-copy genes showing the relationship between strain LZZY-S40 and related taxa. Numbers at nodes are bootstrap values (percentages of 1000 replications); *Nocardia carnea* NBRC 14403^T^ (GCA000308515.1) was used as an outgroup. Bar, 0.10 nucleotide substitutions per site.





**Supplementary Figure S2** Qualitative detection of protease activity of strain LZZY-S40. The strain was cultured on 1% skim milk agar plates and incubated at 28°C for 5 days.

**
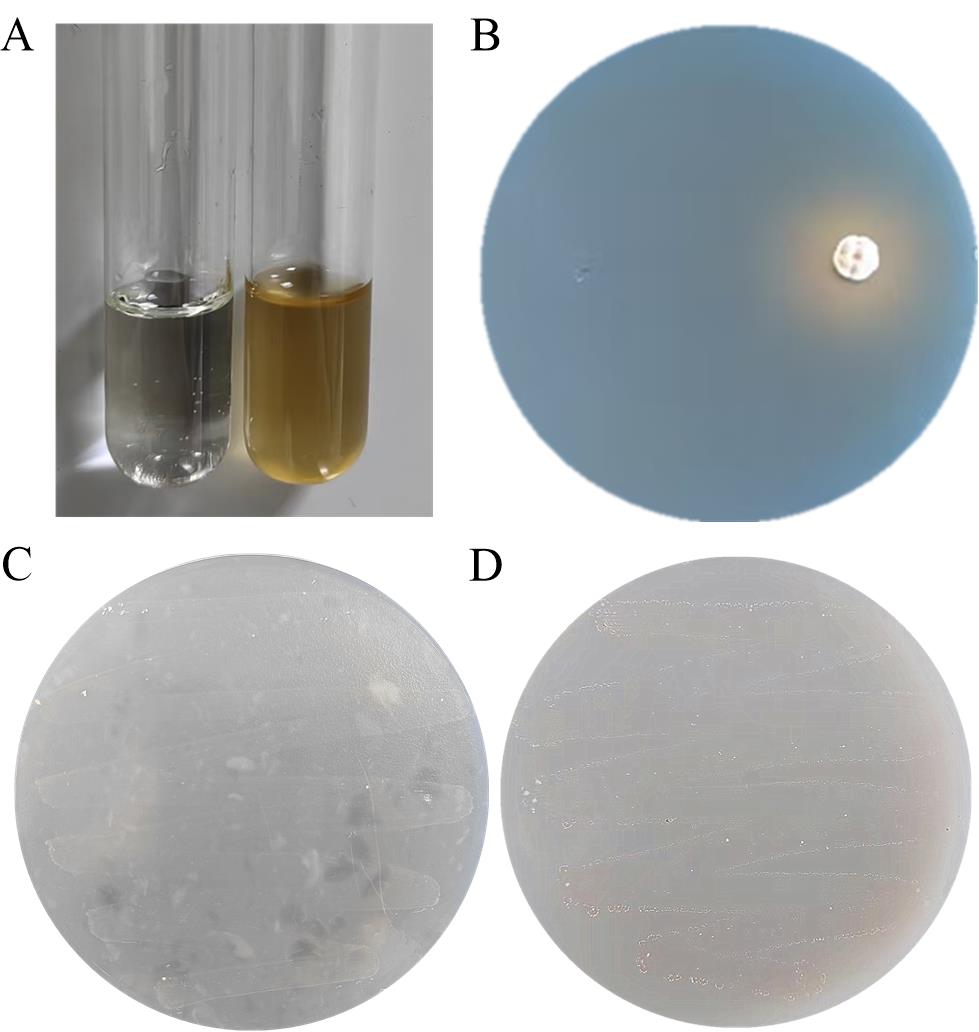
**

**Supplementary** **Figure S3** Qualitative detection of plant growth-promoting traits of strain LZZY-S40. (A) Indole-3-acetic acid (IAA) production, detected using Salkowski reagent. (B) Siderophore production, assessed on CAS agar plates. (C) ACC deaminase activity, evaluated on medium containing ACC as the sole nitrogen source. (D) Nitrogenase activity, determined by growth on nitrogen-free medium.
